# Rainfall has contrasting effects on aquatic and terrestrial environmental DNA recovered from streams

**DOI:** 10.1002/eap.70169

**Published:** 2026-01-11

**Authors:** Olivia P. Reves, Mark A. Davis, Eric R. Larson

**Affiliations:** ^1^ Department of Natural Resources and Environmental Sciences University of Illinois Urbana Illinois USA; ^2^ Illinois Natural History Survey, Prairie Research Institute University of Illinois Champaign Illinois USA

**Keywords:** biodiversity monitoring, biomonitoring, eDNA, Illinois, lotic ecosystems, metabarcoding, precipitation, rainfall, stream flow, Vermilion River

## Abstract

Environmental DNA (eDNA) metabarcoding is increasingly applied to a variety of questions and challenges across basic and applied ecology. Although streams and rivers (i.e., lotic ecosystems) can serve as conveyor belts of both aquatic and terrestrial eDNA from upstream or riparian areas, precipitation can dilute eDNA due to increasing discharge and/or mobilize eDNA into rivers from adjacent terrestrial ecosystems. Previous research has examined eDNA detectability of single species after high flow events, but no studies have compared aquatic and terrestrial communities recovered by eDNA metabarcoding together in response to rainfall. For this study, we used eDNA metabarcoding to sample three rivers before and after precipitation over six sampling events to evaluate if terrestrial eDNA exhibits a mobilization effect and aquatic eDNA exhibits a dilution effect after rainfall. We found that as rainfall increased, terrestrial taxa richness significantly increased and aquatic taxa richness decreased but not significantly. As such, researchers using eDNA metabarcoding from lotic ecosystems to characterize terrestrial communities might not need to avoid, and could even seek out, precipitation events in their sampling design. However, our study should be replicated over more lotic ecosystems and ecoregions and larger gradients of precipitation events.

## INTRODUCTION

Environmental DNA (eDNA) metabarcoding, in which the DNA of entire communities is captured and identified from an environmental sample, is transforming how we understand and monitor the world (Deiner et al., 2017). In the last decade, eDNA metabarcoding has been applied to diverse questions from the early detection of biological invasions (Bell et al., 2024) to metacommunity dynamics (Erős et al., 2024; Zinger et al., 2025). These applications have occurred across marine, freshwater, and terrestrial ecosystems for taxa ranging from bacteria to mammals (Rishan et al., 2025). Despite the ongoing boom in eDNA metabarcoding, many challenges remain on how to best operationalize these complex, emerging methods for basic and applied questions in ecology (Jerde, 2021; Kelly et al., 2024).

One unique aspect of eDNA metabarcoding is that water samples collected from lotic ecosystems (e.g., rivers and streams) detect not only freshwater but also terrestrial taxa of the watersheds they drain (Deiner et al., 2016). This creates opportunities for efficient, low‐effort characterization of terrestrial communities at landscape scales (Reji Chacko et al., 2023) but also challenges in understanding the spatial and temporal dynamics of eDNA information (Barnes & Turner, 2016). For example, precipitation events increase stream discharge and likely dilute eDNA concentrations regardless of origin (Curtis, Tiemann, et al., 2021b; Jane et al., 2015). Conversely, precipitation may also mobilize and transport terrestrial eDNA from forest canopies or soils into lotic ecosystems, counteracting dilution for this source of eDNA (Condachou et al., 2025; Macher et al., 2023; Valentin et al., 2021; Zinger et al., 2025). To date, no studies have investigated the simultaneous effect of precipitation on both aquatic and terrestrial taxa recovered from eDNA metabarcoding in lotic ecosystems.

In this study, we sampled three sites before and after precipitation throughout a year to evaluate how aquatic and terrestrial eDNA metabarcoding results responded to these events. We expected aquatic taxa richness to decline after precipitation due to dilution from increased stream discharge, and terrestrial taxa richness to increase after precipitation due to mobilization into streams from adjacent riparian zones. The results of this study may inform users of eDNA metabarcoding on whether they should seek or avoid precipitation events concurrent with their sampling dependent on their focal taxonomic groups.

## METHODS

We conducted this study in the Vermilion River watershed of Illinois, United States (USA), a tributary to the Mississippi River by the Wabash River. The Vermilion River drains a watershed of primarily row crop agriculture with low human population densities (Hill et al., 2016). We chose three sites associated with United States Geological Survey (USGS) streamgages, which spanned the smaller, minimally forested Salt Fork to the larger, well‐forested Middle Fork, with the North Fork intermediate by both watershed area and natural land cover (Table 1; Appendix S1: Figure S1). We sampled each site six times from October 2023 to June 2024 (Appendix S1: Table S1; Reves & Larson, 2025), pairing eDNA samples for each sampling event as pre‐ and post‐ precipitation days anticipated from weather forecasts. Sampling across seasons was intended to capture gradients of temperature‐dependent activity and site occupancy for a diversity of taxa, including migratory species during autumn and spring. Pre‐precipitation sampling events had generally been dry over the preceding week (Appendix S1: Table S1). We characterized magnitude of precipitation events using gages from the Community Collaborative Rain, Hail, and Snow Network (hereafter precipitation gages). We identified the precipitation gage closest to each of our sites (Table 1; Appendix S1: Figure S1) and downloaded its precipitation data as cumulative cm for the 24 h preceding eDNA sampling. Precipitation gages are measured at 7:00 am every day, and accordingly, our eDNA sampling was conducted during mornings or early afternoons. As stream flow and precipitation of the preceding 24 h were positively correlated (*r* = 0.62), we used 24‐h precipitation as our predictor in subsequent modeling.

**TABLE 1:** Sampling sites (Site) in the Vermilion River watershed of Illinois, United States, with geographic coordinates (Coordinates) in WGS84, their associated USGS streamgage (Stream gage), Strahler stream order (Stream order), upstream drainage area (Area) at the site in square kilometers, forest cover (Forest) in % of the upstream catchment in 2019 (Hill et al., 2016), the precipitation gage (Rain gage) used for precipitation data, distance to the rain gage (Gage distance) in kilometers, and precipitation gage coordinates (Rain coordinates) in WGS84.

Site	Coordinates	Stream gage	Stream order	Area	Forest	Rain gage	Gage distance	Rain coordinates
Salt Fork	40.1495, −88.0336	03336900	5	347.06	7.72	IL‐CP‐137: St. Joseph 1.1 ENE	3.2	40.1176, −88.0213
North Fork	40.2658, −87.6424	03338780	7	678.58	37.51	IL‐VR‐4: Henning 3.4 SSE	1.6	40.2647, −87.6656
Middle Fork	40.1369, −87.7458	03336645	6	1118.87	71.04	IL‐VR‐45: Fithian 2.6 ESE	6.7	40.0990, −87.8297

*Note*: Hydrography data were taken from the National Hydrography Database. Streamgage data from USGS were accessed at https://waterdata.usgs.gov/nwis/rt and precipitation gage data at https://www.cocorahs.org.

At each site and sampling event, we took three 1‐L water samples in plastic bottles (Nalgene, USA), distributed as one from the left bank, one from the right bank, and one from mid‐channel. We recorded water temperature (in degrees Celsius), pH, and total dissolved solids (ppm) with a multiparameter probe (JulyPanny, China) and turbidity (NTU) with a turbidity meter (Sper Scientific, USA) at mid‐channel. Sites were slightly basic, generally clear, with total dissolved solids ranging from 200 to 400 ppm (Reves & Larson, 2025). Water samples were transported in a cooler on ice to our laboratory (Curtis, Larson, & Davis, 2021a) where they were immediately filtered (<3 h) onto 1.0–1.2‐μM cellulose nitrate membrane filters (Whatman, UK) using a vacuum pump (Steriltech, USA). After at least 2 weeks in room temperature cetyltrimethylammonium bromide buffer, we extracted DNA from the filters using a phenol‐chloroform‐isoamyl precipitation method (García et al., 2024).

We targeted terrestrial and aquatic vertebrates as taxonomic priorities for management agencies in our study region using a two‐step PCR metabarcoding library preparation protocol (Moss et al., 2022) to target both a 73–110 bp region of the mitochondrial 12S gene with vertebrate‐specific primers (Riaz et al., 2011) and a 313 bp region of the mitochondrial Cytochrome c Oxidase subunit I (COI) gene with universal primers (Appendix S1: Table S2; Leray et al., 2013). Primers were chosen due to good performance by our laboratory in past applications for vertebrate communities (Tetzlaff et al., 2024). Both primer sets were modified with Illumina overhang adapter sequences and used for the first PCR, where we ran triplicate 25‐μL reactions. For the second PCR, we assigned unique barcodes to each sample with Illumina UD Indexes (Integrative DNA Technologies, USA), and we ran duplicate 20‐μL reactions. Negative PCR controls (molecular grade water) were run on all PCR plates. We confirmed amplification via gel electrophoresis and performed bead cleanups with SPRISelect beads (Beckman Coulter, USA). We quantified the DNA concentration for each sample (in nanograms per microliter) via the Qubit dsDNA HS kit (Invitrogen, USA) and normalized libraries. Next‐generation sequencing was completed on the Illumina NovaSeq X Plus at the W.M. Keck Core Sequencing Facility in the Roy J. Carver Biotechnology Center, University of Illinois, Urbana‐Champaign.

We trimmed demultiplexed sequences using cutadapt (Martin, 2011) for both the 12S and COI primer sets. We filtered, merged, and checked demultiplexed sequences for chimeras using the DADA2 denoising plugin (Callahan et al., 2016) within QIIME2 v 2024.10 (Bolyen et al., 2019). We assigned taxonomic classifications to each sequence using the Basic Local Alignment Search Tool (BLAST) within Geneious Prime (Geneious Prime 2024.0.5; https://www.geneious.com) and a custom species list for vertebrates of Illinois from Illinois Natural History Survey species checklists (https://inhs.illinois.edu/resources/biological-collections/). At this time, sequences assigned to domesticated, farmed, and vertebrate taxa believed to derive from laboratory contamination (e.g., house mouse *Mus musculus*) were discarded. We converted raw read counts of recovered vertebrate taxa into binary presences (1) and absences (0) due to the challenges of accurately attaining relative abundance from eDNA metabarcoding results (Deiner et al., 2017). We refer to taxa, rather than species, richness because some classifications were to the genus or family level. Lastly, we removed four samples (Appendix S1) where at least one paired site detected little to no taxa, possibly because of potential laboratory or PCR amplification failure. This resulted in 32 samples (16 pairs) across six sampling events. More detailed methods across eDNA sampling, metabarcoding library preparation, and bioinformatics can be found in Appendix S1.

We built separate models for obligate aquatic (i.e., fish) and terrestrial (i.e., mammal, bird, amphibian, reptile) vertebrate taxa richness, while acknowledging that our terrestrial taxa have varying dependencies on lotic ecosystems. In each model, taxa richness was the response variable regressed against the preceding 24‐h precipitation and water temperature as fixed effects. Water temperature was included to represent potential differences in taxa richness or activity across seasons. Random effects included site (Salt Fork, Middle Fork, North Fork) to account for differences among sampling locations and sampling event to account for the pairing of pre‐ and post‐ precipitation samples (Appendix S1: Table S1). We ran models using the “nlme” package (Pinheiro et al., 2025) in *R* version 4.4.2 (R Core Team, 2022). Normality of model residuals was confirmed with Shapiro–Wilk tests.

## RESULTS

**FIGURE 1:**
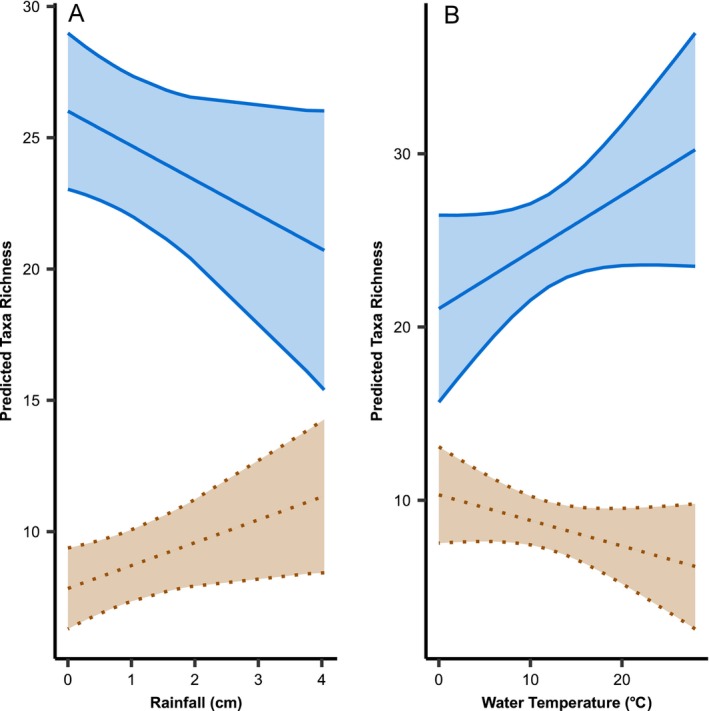
Linear mixed model predictions for aquatic (blue solid) and terrestrial taxa richness (brown dotted) in response to (A) rainfall (in centimeters) and (B) water temperature (in degrees Celsius) with 95% CIs.

We identified 118 unique taxa across five vertebrate classes (Appendix S1: Figures S2 and S3; Reves & Larson, 2025). Precipitation had a positive, significant effect on terrestrial taxa richness (estimate = 0.87, SE = 0.38, *t* = 2.28, χ^2^ (1) = 5.21, *p* = 0.022), but no significant effect on aquatic taxa richness (estimate = −1.31, SE = 0.68, *t* = −1.93, χ^2^ (1) = 3.72, *p* = 0.054; Figure 1). Water temperature had no significant effect on terrestrial taxa richness (estimate = −0.15, SE = 0.10, *t* = −1.53, χ^2^ (1) = 2.36, *p* = 0.125) or aquatic taxa richness (estimate = 0.33, SE = 0.18, *t* = 1.81, χ^2^ (1) = 3.27, *p* = 0.07; Figure 1). The model of terrestrial taxa richness had a marginal *R* ^2^ of 0.20 and a conditional *R* ^2^ of 0.49, whereas the model of aquatic taxa richness had a marginal *R* ^2^ of 0.24 and a conditional *R* ^2^ of 0.46.

## DISCUSSION

We found opposing effects of precipitation on terrestrial relative to aquatic taxa richness recovered by eDNA metabarcoding from lotic ecosystems, with terrestrial taxa richness increasing post‐precipitation and aquatic taxa richness unaffected. Notably, a weak, negative effect of precipitation on aquatic taxa richness might be identified with greater statistical power than our replication here allowed. We found no significant effects of water temperature on taxa for either terrestrial or aquatic organisms. Disparate effects of precipitation events on terrestrial and aquatic taxa richness for eDNA recovered from lotic ecosystems may inform sampling strategies using metabarcoding methods and interpretations of community structure or change over environmental gradients (Erős et al., 2024). For example, surveys specifically seeking to use rivers and streams as conveyor belts of terrestrial biodiversity information (Deiner et al., 2016) might benefit from sampling after precipitation events, potentially with little effect on aquatic communities recovered (Curtis, Tiemann, et al., 2021b; Osathanunkul & Suwannapoom, 2024). We recommend other researchers replicate our findings across different ecosystems or larger gradients of precipitation events to further validate these results.

While a recent study detected more semiaquatic and terrestrial species in rainy seasons versus dry seasons using eDNA metabarcoding (Condachou et al., 2025), our study is the first to document a positive effect of precipitation events on terrestrial eDNA in streams and rivers. Our result is consistent with studies that have found rainfall removes DNA from surfaces (Johnson et al., 2019; Valentin et al., 2021) and that rainwater contains DNA from adjacent vegetation (Macher et al., 2023; Zinger et al., 2025). Our results emphasize that the life cycle of eDNA does not end when rainfall washes it away, but instead precipitation increases detectability of some terrestrial taxa in aquatic environments post‐storm. Further, we found a stronger effect of precipitation on mobilization of terrestrial eDNA, revealed by increased taxa richness post‐precipitation, than dilution from increased discharge. This result was surprising, as past single‐species eDNA studies have routinely found significant effects of dilution on eDNA concentrations or detectability for fish or freshwater invertebrates (Curtis, Tiemann, et al., 2021b; Osathanunkul & Suwannapoom, 2024; but see George et al., 2024). We may have recovered a stronger or significant negative effect of precipitation on dilution of aquatic eDNA if we sampled with higher replication, or potentially over a larger gradient of precipitation events. The precipitation events we were able to sample were modest, with a mean of 0.88 cm over a 24‐h period (0.00 cm minimum to 4.04 cm maximum), whereas heavier storm events and higher stream discharge may have had different effects on both terrestrial and aquatic eDNA taxa richness.

We may have also been under‐powered to detect an effect of temperature on our eDNA metabarcoding results, although increasing temperature did correspond to lower terrestrial taxa richness and higher aquatic taxa richness in weak and nonsignificant relationships. Increased metabolic activity with warmer temperatures can result in increased shedding of eDNA by fish in lotic ecosystems (Lacoursière‐Roussel et al., 2016), whereas warmer temperatures might primarily be affecting terrestrial eDNA that originates outside of streams and rivers through degradation in these ecosystems (Barnes & Turner, 2016). Again, increased replication in other ecosystems might resolve the weak relationships recovered here. It would also be valuable to replicate our study in different ecoregions and stream ecosystems. For example, stream size or discharge affects eDNA transport and persistence dynamics (Shogren et al., 2017), and this work could be replicated in both smaller and larger ecosystems than the wadeable, mid‐order streams sampled here. Extending this idea, replication across stream size could allow researchers to determine whether small or large streams are better conveyor belts of terrestrial biodiversity information in the context of theories like the River Continuum Concept (Vannote et al., 1980).

If the results of our study persist over time, researchers using eDNA metabarcoding to recover upstream terrestrial communities from lotic ecosystems would be well advised to sample after moderate precipitation events, which seemingly mobilize DNA from adjacent riparian environments into streams and rivers. These precipitation events can, of course, be difficult to anticipate; in our case, forecast precipitation did not always occur or often occurred at lower magnitudes than desired for the study. Regardless, precipitation events might not be specifically avoided in terrestrial eDNA studies using lotic ecosystems, in contrast to recommended avoidance of the dilution effect when targeting obligate aquatic taxa (Curtis, Tiemann, et al., 2021b; Osathanunkul & Suwannapoom, 2024). Cumulatively, our study reveals that much remains to be discovered about the ecology of eDNA at the interface of organisms and their environment (Barnes & Turner, 2016; Newton et al., 2025).

## CONFLICT OF INTEREST STATEMENT

The authors declare no conflicts of interest.

## Supporting information


Appendix S1.

## Data Availability

Data (Reves & Larson, 2025) are available in the Illinois Data Bank at https://doi.org/10.13012/B2IDB-9609945_V1.
