# Supplementary material for: Rainfall has contrasting effects on aquatic and terrestrial environmental DNA recovered from streams
Source: Ecol Appl. 2026 Jan 11;36(1):e70169. doi: 10.1002/eap.70169 (PMC12792245; doi:10.1002/eap.70169)
Supplement: Supplementary file 1 — Appendix S1. [file EAP-36-e70169-s001.pdf]

Appendix S1

Rainfall has contrasting effects on aquatic and terrestrial environmental DNA recovered from streams

Olivia P. Reves, Mark A. Davis, Eric R. Larson

Ecological Applications

Contents

Section S1

Table S1. Detailed sampling event data.

Table S2. Information about primers.

Table S3. Sequencing summary statistics.

Figure S1. Sampling site map.

Figure S2. Heatmap for aquatic taxa detected in this study.

Figure S3. Heatmap for terrestrial taxa detected in this study.

Section S1

Environmental DNA sampling and processing

We sampled three sites associated with United States Geological Survey (USGS) streamgages in the Vermilion River watershed of east-central Illinois, United States over four seasons across a forest cover gradient (Figure S1). For each eDNA sampling event, we lowered a bucket from the bridge into the stream at the study site to fill three sterile 1 L plastic bottles (Nalgene, USA), sampling across a lateral transect of the stream channel. Before every use, the 1 L plastic bottles were decontaminated with 10 or 50% bleach and exposed to UV light for at least 20 minutes. We used separate buckets for each site and decontaminated them with 10 or 50% bleach before and after use, rinsing them with stream water at the site before sampling. We intended to sample sites immediately before and during the rising limb of peak discharge associated with a rain event. For every site, we transported and processed one field blank of 1 L of deionized water along with field samples. At each sampling site and event, we recorded water temperature, pH, and total dissolved solids with a multiparameter probe (JulyPanny, China), and turbidity with a turbidity meter (Sper Scientific, USA).

Sample processing was performed under sterile conditions (all surfaces cleaned with 10 or 50% bleach) in workspaces at the Illinois Natural History Survey's Collaborative Conservation Genomics Laboratory physically isolated from rooms with high copy DNA. Immediately following a sampling event, we filtered field samples onto 1.0 µM cellulose nitrate membrane filters (Whatman™, UK) using a vacuum pump (Steriltech, USA), also filtering and processing a filtration blank during this time. Filtration replicates were utilized as needed if the sample was dirty or if the filter ripped to filter the entire 1 L. Samples were kept in cetyltrimethylammonium bromide (CTAB) buffer at room temperature for a minimum of two weeks before being stored in a –20°C freezer until DNA extraction. The samples collected on April 2, 2024 were filtered onto 1.2 µM cellulose nitrate membrane filters due to a shortage of 1.0 µM filters at the time (Whatman™, UK). Because previous literature has found no significant effects of cellulose nitrate pore size (0.45-1.2 µM) on DNA yield for metabarcoding (Li et al., 2018), our samples filtered with 1.2 µM filters should exhibit similar DNA yield as our samples filtered with 1.0 µM filters. After at least two weeks in room temperature CTAB buffer, we extracted DNA from the filters using a phenol-chloroform-isoamyl precipitation method (García et al. 2024). In tandem with CTAB buffer as a storage method, our extraction protocol is broadly resistant to PCR inhibition (Hunter et al., 2019). At this step, extraction blanks were processed alongside field samples, and filtration replicates were pooled. In total, we processed 108 field samples, 36 field blanks, and 12 filtration blanks.

Environmental DNA Metabarcoding

We used a two-step PCR metabarcoding approach to amplify extracted DNA via two primers that target vertebrates to account for potential primer biases (Moss et al. 2022). We

targeted a 73-110 bp region of the mitochondrial 12S gene by using vertebrate-specific primers established by Riaz et al. (2011) and a 313 bp region of the mitochondrial Cytochrome c Oxidase subunit I (COI) gene (Leray et al. 2013; Table S2). Both primer sets were modified with Illumina overhang adapter sequences and used for the first PCR (PCR1). PCR1 reactions were performed in triplicates and a 25 µL reaction volume composed of 12.5 µL of Q5® High-Fidelity 2X Master Mix (New England Biolabs, USA), 0.5 µL of bovine serum albumin (BSA; Thermofisher Scientific, USA), 6.0 µL of molecular grade water, 1.0 µL of forward primer (10 µM), and 1.0 of reverse primer (10 µM). Negative PCR controls (molecular grade water) were run in triplicate on all PCR1 plates. For the 12s primers, the PCR1 thermocycling conditions were as follows: initial denaturation at 98°C for 5 min; 35 cycles of: 98°C, 58°C, 72°C for 10s, 30s, 30s; extension at 72°C for 7 min; storage at 4°C for ∞. For the COI primers, the PCR1 thermocycling conditions were as follows: initial denaturation at 95°C for 5 min; 16 cycles of: 95°C, 62°C, 72°C for 10s, 30s, 60s; 25 cycles of: 95°C, 46°C, 72°C for 10s, 30s, 60s; extension at 72°C for 7 min; storage at 4°C for ∞. Following PCR1, triplicate samples were pooled and successful amplification was visualized via gel electrophoresis. To remove excess primer, PCR1 products were cleaned with SPRISelect beads (Beckman Coulter, USA) at a volume of 55 µL per sample and verified on a gel. For the second PCR (PCR2), unique barcodes were added to each sample with Illumina UD Indexes (Integrative DNA Technologies, USA).

PCR2 reactions for each primer set were performed in duplicate 20 µL reactions consisting of 10 µL of Q5® High-Fidelity 2X Master Mix, 2 µL of index primers (10 µM), 5.0 µL of molecular grade water, and 3 µL of DNA (PCR1 product). Negative PCR controls (molecular grade water) were run in duplicate on all PCR2 plates. The PCR2 thermocycling conditions were identical for both primer sets: initial denaturation at 98°C for 3 min; 10 cycles of

98°C, 55°C, 72°C for 30s, 30s, 30s; extension at 72°C for 5 min; storage at 4°C for ∞. Following PCR2, duplicate samples were pooled and successful amplification was confirmed via gel electrophoresis. A second bead cleanup was performed with SPRISelect beads to remove any excess primer at a minimum volume of 21 μL per sample and verified via gel electrophoresis. The DNA concentration for each sample was quantified (ng/μL) via Qubit dsDNA HS kit (Invitrogen, U.S.). Sub-libraries were created after samples were normalized and pooled per plate based on calculated DNA concentrations. A third bead cleanup was performed with SPRISelect beads for each sub-library and verified via gel electrophoresis. Sub-libraries were then normalized and pooled, yielding two completed libraries (12s and COI). However, due to the total quantity of samples (n=476) exceeding the maximum allowed on a single sequencing run (n=384), two separate sequencing runs were performed per primer set. The first sequencing run consisted of 380 samples, while the second sequencing run consisted of the remaining 96 samples. In total, four sequencing runs were completed. Prior to sequencing, QA/QC was completed on an Aglient Tape Station at the W.M. Keck Core Sequencing Facility at the Roy J. Carver Biotechnology Center, University of Illinois, Urbana-Champaign. Finally, all samples were sequenced on an Illumina NovaSeq X Plus.

Bioinformatics & Statistical Analyses

Demultiplexed sequences were trimmed using cutadapt (Martin 2011) for both the 12S and COI primer sets. Demultiplexed sequences were filtered, merged and checked for chimeras using the DADA2 denoising plugin (Callahan et al. 2016) within QIIME2 v 2024.10 (Bolyen et al. 2019). For both primer sets, default settings were used with the exception of the truncating length parameter. For the 12s primer set, '*p-trunc-len-f-73*' and '*p-trunc-len-r-73*' was used. It is

important to note here that because the COI primer set has a fragment size larger than 150 bp, COI sequences had to be uploaded into QIIME2 separately. For the COI (forward) primer set, '*p-trunc-len 81*' was used and for the COI (reverse) primer set, '*p-trunc-len 100*' was used.

We assigned taxonomic classifications to each sequence using the Basic Local Alignment Search Tool (BLAST) within Geneious Prime (Geneious Prime® 2024.0.5; https://www.geneious.com) and a custom species list for vertebrates of Illinois using Illinois Natural History Survey species checklists (https://inhs.illinois.edu/resources/biological-collections/). First, we filtered amplicon sequencing variants (ASV's) using the Entrez Query "Vertebrata [Organism] NOT Primate [Organism]" into "hit or no hit" categories. Sequences that did not match the Entrez Query or did not have a taxonomic assignment were discarded. Then, we used BLAST on the sequences from the "hits." We set a pairwise identity threshold of >97% and cross-referenced our custom species list to assign the most plausible finest taxonomic classification, only retaining taxa at a minimum family level. On multiple occasions during this step, we observed muskrat (*Ondatra zibethicus*) and American mink (*Neogale vison*) sequences to perfectly match each other (identical pairwise identity and E-value). We created a phylogenetic tree within Geneious Prime to inspect the relationship between *O. zibethicus* and *N. vison* reference sequences. One of the top *N. vison* sequences (Name in Geneious Prime: MT874477) was more closely related to *O. zibethicus*, so for these instances, we classified all ASV's as *O. zibethicus*. At this time, sequences assigned to domesticated or farm vertebrate species were discarded. Additionally, taxa believed to derive from laboratory contamination, such as rainbow smelt (*Osmerus mordax*) and mottled sculpin (*Uranidea bairdii*), were discarded. To address further contamination, the maximum read count from possible contaminants in control blanks was subtracted from sample read counts. To account for potential

tag jumps, we set a 0.01% frequency of occurrence threshold (Schnell et al. 2015). This permissive threshold is plausible for this study due to the dominance of fish sequences within our samples. At this time, we consolidated read counts (12s, COI Forward, COI Reverse) into one spreadsheet for future downstream analyses.

Prior to any statistical analyses, we accounted for taxonomic redundancy (Reji Chacko et al. 2023; Marques et al. 2020). A genus or family assignment was only kept within the dataset if there were no species already belonging to the rank. We ensured the higher-level groups only included species that were not already counted under their corresponding groups; for example, we detected yellow bullhead (*Ameiurus natalis)* to species level, but black bullhead (*Ameiurus melas)* and brown bullhead (*Ameiurus nebulosus)* were only detected to genera (*Ameiurus spp.).* Ultimately, each "taxa" in our statistical analyses represents one, or a set, of unique species with no nested taxa resulting in overrepresented taxonomic richness.

We removed four samples (6_25_24_NF and 6_26_24_NF; 10_28_23_NF and 10_29_23_NF) where at least one paired site detected little to no taxa, possibly because of potential laboratory or PCR amplification failure. This resulted in 32 samples (16 pairs) across six sampling events.

Sequencing Summary Statistics

All summary sequencing statistics specific to this study are reported in Table S3. After filtering for human, non-vertebrate taxa, domestic vertebrate species and contamination from the laboratory, gizzard shad (*Dorosoma cepedianum*) was the most common contaminant, so it was taken out of 31 out of 108 samples within the 12s reads. Overall contamination levels between both primer sets remained low. We identified 118 unique taxa across classes Actinopterygii,

Aves, Amphibia, Mammalia, and Reptilia comprising of 26 orders, 49 families, 86 genera, and 99 species (Figure S2, Figure S3, Reves and Larson, 2025). While 84% of taxa were identified to species, some were only assigned classification to the genus or family level, like *Anaxyrus spp*. or Emberizidae. Actinopterygii had the most taxa (62 taxa, 8 orders, 15 families, 39 genera, 53 species) followed by Aves (29 taxa, 9 orders, 18 families, 26 genera, 21 species), Mammalia (17 taxa, 6 orders, 9 families, 15 genera, 17 species), Amphibia (7 taxa, 2 orders, 4 families, 4 genera, 5 species) and Reptilia (3 taxa, 1 order, 3 families, 3 genera, 3 species). Among fish, the most common order was Cypriniformes comprising 44% of taxa, followed by Perciformes comprising 35% of taxa. Among terrestrial and semi-aquatic vertebrates, the most common orders were Passeriformes (11 taxa), and Rodentia (9 taxa).

REFERENCES

Bolyen, Evan, Jai Ram Rideout, Matthew R. Dillon, Nicholas A. Bokulich, Christian C. Abnet, Gabriel A. Al-Ghalith, Harriet Alexander, et al. “Reproducible, Interactive, Scalable and Extensible Microbiome Data Science Using QIIME 2.” *Nature Biotechnology* 37, no. 8 (August 2019): 852–57. https://doi.org/10.1038/s41587-019-0209-9.

Callahan, Benjamin J, Paul J McMurdie, Michael J Rosen, Andrew W Han, Amy Jo A Johnson, and Susan P Holmes. “DADA2: High-Resolution Sample Inference from Illumina Amplicon Data.” *Nature Methods* 13, no. 7 (July 2016): 581–83. https://doi.org/10.1038/nmeth.3869.

García, Samantha M., Chan Lan Chun, Josh Dumke, Gretchen J. A. Hansen, Kathleen B. Quebedeaux, Christopher Rounds, Anna Totsch, and Eric R. Larson. “Environmental DNA Storage and Extraction Method Affects Detectability for Multiple Aquatic Invasive Species.” *Environmental DNA* 6, no. 3 (May 2024): e557. https://doi.org/10.1002/edn3.557

Hunter, Margaret E., Jason A. Ferrante, Gaia Meigs-Friend, and Amelia Ulmer. “Improving eDNA Yield and Inhibitor Reduction through Increased Water Volumes and Multi-Filter Isolation Techniques.” Scientific Reports 9, no. 1 (2019): 5259. https://doi.org/10.1038/s41598-019-40977-w.

Leray, Matthieu, Joy Y Yang, Christopher P Meyer, Suzanne C Mills, Natalia Agudelo, Vincent Ranwez, Joel T Boehm, and Ryuji J Machida. “A New Versatile Primer Set Targeting a Short Fragment of the Mitochondrial COI Region for Metabarcoding Metazoan Diversity: Application for Characterizing Coral Reef Fish Gut Contents.” *Frontiers in Zoology* 10, no. 1 (2013): 34. https://doi.org/10.1186/1742-9994-10-34.

Li, Jianlong, Lori-Jayne Lawson Handley, Daniel S. Read, and Bernd Hänfling. "The Effect of Filtration Method on the Efficiency of Environmental DNA Capture and Quantification via Metabarcoding." Molecular Ecology Resources 18, no. 5 (2018): 1102–14. https://doi.org/10.1111/1755-0998.12899.

Marques, Virginie, Pierre-Édouard Guérin, Mathieu Rocle, Alice Valentini, Stéphanie Manel, David Mouillot, and Tony Dejean. "Blind Assessment of Vertebrate Taxonomic Diversity across Spatial Scales by Clustering Environmental DNA Metabarcoding Sequences." *Ecography* 43, no. 12 (December 2020): 1779–90. https://doi.org/10.1111/ecog.05049.

Martin, Marcel. "Cutadapt Removes Adapter Sequences from High-Throughput Sequencing Reads." *EMBnet.Journal* 17, no. 1 (May 2, 2011): 10. https://doi.org/10.14806/ej.17.1.200.

Moss, Wynne E., Lynsey R. Harper, Mark A. Davis, Caren S. Goldberg, Matthew M. Smith, and Pieter T. J. Johnson. "Navigating the Trade-offs between Environmental DNA and Conventional Field Surveys for Improved Amphibian Monitoring." *Ecosphere* 13, no. 2 (February 2022): e3941. https://doi.org/10.1002/ecs2.3941.

Reji Chacko, Merin, Florian Altermatt, Fabian Fopp, Antoine Guisan, Thomas Keggin, Arnaud Lyet, Pierre-Louis Rey, et al. "Catchment-Based Sampling of River eDNA Integrates Terrestrial and Aquatic Biodiversity of Alpine Landscapes." *Oecologia* 202, no. 4 (August 2023): 699–713. https://doi.org/10.1007/s00442-023-05428-4.

Reves, Olivia, and Eric Larson. "Data for 'Environmental DNA Metabarcoding of Vertebrates from Central Illinois, United States, 2023-2024.'" University of Illinois Urbana-Champaign, 2025. https://doi.org/10.13012/B2IDB-9609945_V1.

Riaz, Tiayyba, Wasim Shehzad, Alain Viari, François Pompanon, Pierre Taberlet, and Eric Coissac. "ecoPrimers: Inference of New DNA Barcode Markers from Whole Genome Sequence Analysis." *Nucleic Acids Research* 39, no. 21 (November 1, 2011): e145–e145. https://doi.org/10.1093/nar/gkr732.

Schnell, Ida Bærholm, Kristine Bohmann, and M. Thomas P. Gilbert. "Tag Jumps Illuminated – Reducing Sequence-to-sample Misidentifications in Metabarcoding Studies." *Molecular Ecology Resources* 15, no. 6 (November 2015): 1289–1303. https://doi.org/10.1111/1755-0998.12402.

TABLES

Table S1. Sampling events by date and flow status (before or after precipitation events), grouped by river with rainfall recorded at 7:00am for the preceding 24 hours at the nearest Community Collaborative Rain Hail and Snow Network precipitation gage and water temperature (°C) at the time of sampling.

Sampling Event	Date	Flow Status	River	Rainfall (cm)	Water Temperature (°C)
10_28_23_MF	10_28_23	Before	Middle Fork	0.71	13.8
10_28_23_NF	10_28_23	Before	North Fork	0.51	12.8
10_28_23_SF	10_28_23	Before	Salt Fork	1.78	11.7
10_29_23_MF	10_29_23	After	Middle Fork	1.55	10.8
10_29_23_NF	10_29_23	After	North Fork	2.34	11.3
10_29_23_SF	10_29_23	After	Salt Fork	1.7	9.9
11_30_23_MF	11_30_23	Before	Middle Fork	0	2.9
11_30_23_NF	11_30_23	Before	North Fork	0	0.8
11_30_23_SF	11_30_23	Before	Salt Fork	0	2.6
12_01_23_MF	12_01_23	After	Middle Fork	1.78	3.9
12_01_23_NF	12_01_23	After	North Fork	1.93	3.1
12_01_23_SF	12_01_23	After	Salt Fork	1.7	5.5
03_04_24_MF	03_04_24	Before	Middle Fork	0	14
03_04_24_NF	03_04_24	Before	North Fork	0	13.8
03_04_24_SF	03_04_24	Before	Salt Fork	0	16
03_05_24_MF	03_05_24	After	Middle Fork	0.13	10.9
03_05_24_NF	03_05_24	After	North Fork	0.28	11.4
03_05_24_SF	03_05_24	After	Salt Fork	0.03	10.6
03_25_24_MF	03_25_24	Before	Middle Fork	0	8.6
03_25_24_NF	03_25_24	Before	North Fork	0	8.1
03_25_24_SF	03_25_24	Before	Salt Fork	0	8.8
03_26_24_MF	03_26_24	After	Middle Fork	0.84	9.0
03_26_24_NF	03_26_24	After	North Fork	0.94	8.5
03_26_24_SF	03_26_24	After	Salt Fork	0.48	8.7
03_31_24_MF	03_31_24	Before	Middle Fork	0	13.8
03_31_24_NF	03_31_24	Before	North Fork	0	12.6
03_31_24_SF	03_31_24	Before	Salt Fork	0	13.5
04_02_24_MF	04_02_24	After	Middle Fork	3.84	13.4
04_02_24_NF	04_02_24	After	North Fork	3.76	11.9
04_02_24_SF	04_02_24	After	Salt Fork	4.04	11.9

Table S1 (Continued).

Sampling Event	Date	Flow Status	River	Rainfall (cm)	Water Temperature (°C)
06_25_24_MF	06_25_24	Before	Middle Fork	0	26.5
06_25_24_NF	06_25_24	Before	North Fork	0	25.7
06_25_24_SF	06_25_24	Before	Salt Fork	1.04	24.4
06_26_24_MF	06_26_24	After	Middle Fork	0.53	25.0
06_26_24_NF	06_26_24	After	North Fork	0.41	23.9
06_26_24_SF	06_26_24	After	Salt Fork	1.04	24.8

Table S2. Primers used in this study with index adapter (bold) and references.

Primer	Sequence	Reference
12s-V5_forward	**TCGTCGGCAGCGTCAGATGTGTATAAGAGACAG**-TAGAACAGGCTCCTCTAG	Riaz et al., 2011
12s-V5_reverse	**GTCTCGTGGGCTCGGAGATGTGTATAAGAGACAG**-TTAGATACCCCACTATGC	Riaz et al., 2011
mlCOIintF_forward	**TCGTCGGCAGCGTCAGATGTGTATAAGAGACAG**-GGWACWGGWTGAACWGTWTAYCCYCC	Leray et al., 2013
HCO2198_reverse	**GTCTCGTGGGCTCGGAGATGTGTATAAGAGACAG**-TAAACTTCAGGGTGACCAAAAAATCA	Leray et al., 2013
Index 1 (i7)	**CAAGCAGAAGACGGCATACGAGAT[i7-10bp]**GTCTCGTGGGCTCGG	IDT-Illumina UD Indexes
Index 2 (i5)	**AATGATACGGCGACCACCGAGATCTACAC[i5-10bp]**TCGTCGGCAGCGTC	IDT-Illumina UD Indexes

Table S3. Sequencing summary statistics for all primer sets used in this study. Statistics include the total number of raw sequencing reads (Raw Reads), the total number of filtered reads (Filtered Reads), the total number of filtered, and non-chimeric reads (Non-chimeric Reads) and the total number of amplicon sequencing variants (ASV's) assigned to taxa regardless of taxonomy level (ASV's).

**Primer**	**Raw Reads**	**Filtered Reads**	**Non-chimeric Reads**	**ASV'S**
12s-V5	77,523,012	77,088,403	60,250,839	1,150
mlCOIintF_forward	92,552,958	91,764,938	87,672,330	9,566
HCO2198_reverse	92,552,958	91,977,824	73,450,638	10,921

FIGURES

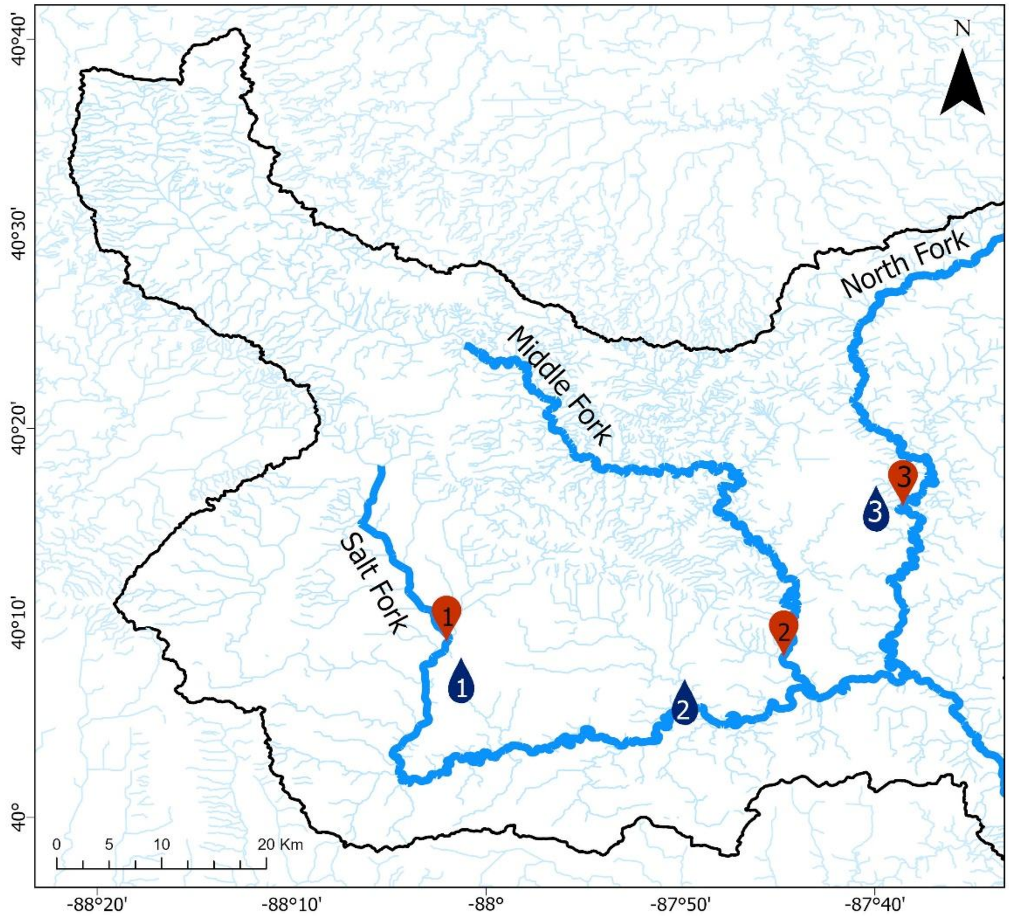

Figure S1. Three sampling sites in the Vermilion River watershed, Illinois, United States (red) at USGS streamgages (https://waterdata.usgs.gov/nwis/rt) and the associated precipitation gages (blue; https://www.cocorahs.org) paired by numbers (1, 2, 3). The eastern border of the map is the border between Illinois and Indiana. Flow lines from USGS (National Hydrography Database).

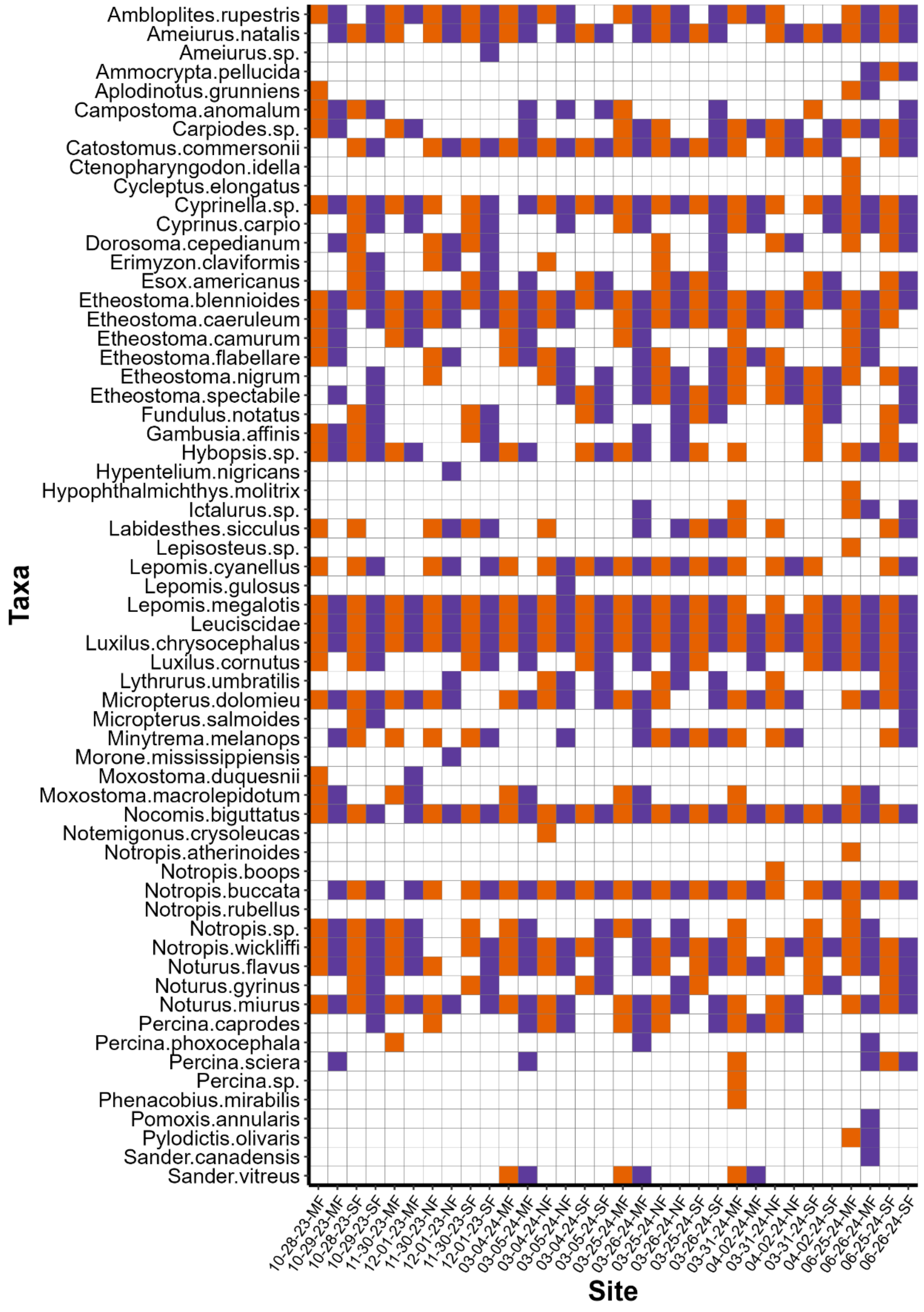

Figure S2. Recovered obligate aquatic taxa (fish) in alphabetical order by sampling events and sites paired before (orange) or after (purple) a precipitation event. .

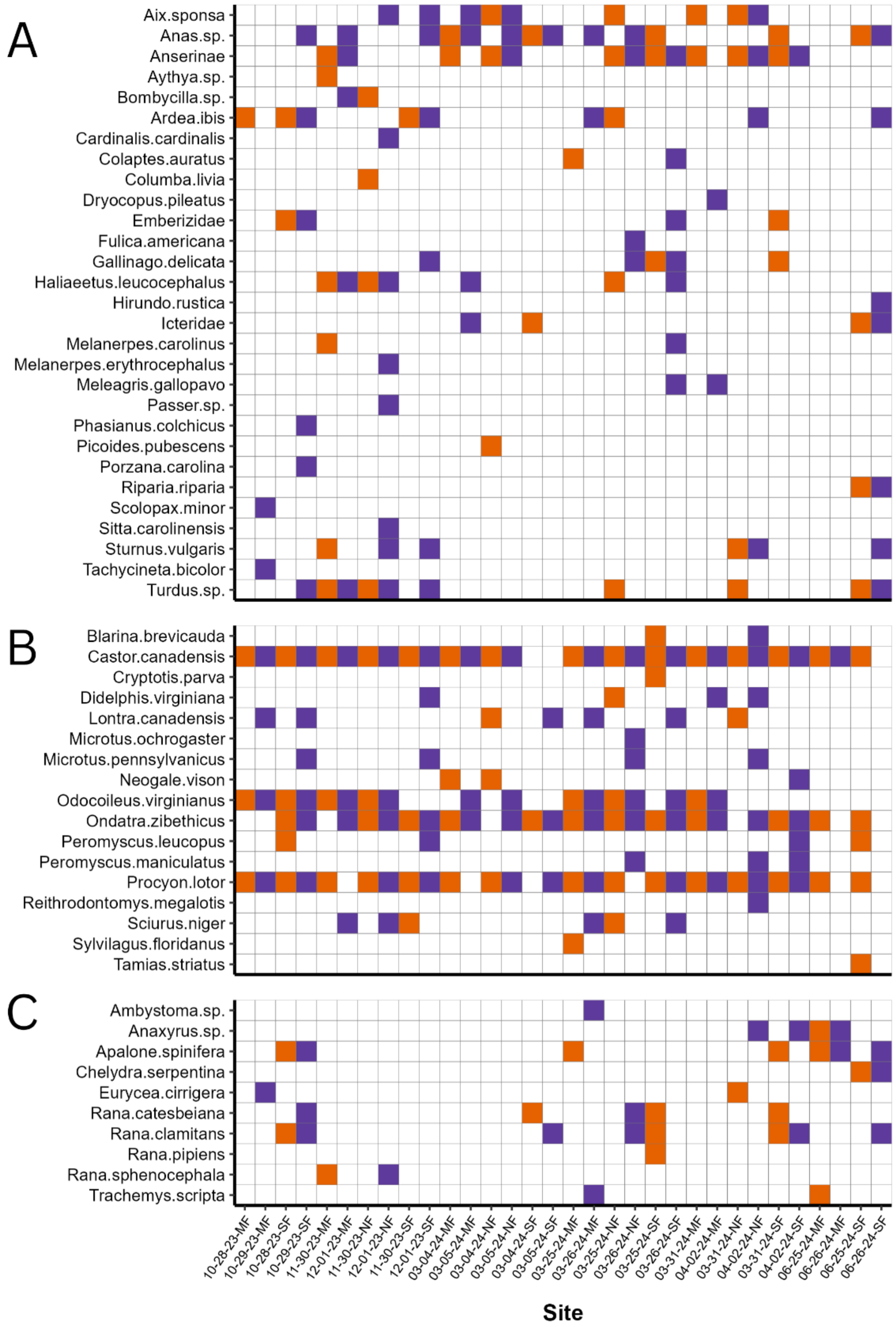

Figure S3. Recovered terrestrial taxa categorized by class: Aves (A), Mammalia (B), Reptilia and Amphibia (C) in alphabetical order by sampling events and sites paired before (orange) or after (purple) a precipitation event.
